# Symptomatic malaria diagnosis overestimate malaria prevalence, but underestimate anaemia burdens in children: results of a follow up study in Kenya

**DOI:** 10.1186/1471-2458-14-332

**Published:** 2014-04-09

**Authors:** Joseph K Choge, Ng’wena G Magak, Willis Akhwale, Julius Koech, Moses M Ngeiywa, Elijah Oyoo-Okoth, Fabian Esamai, Odipo Osano, Christopher Khayeka-Wandabwa, Eliningaya J Kweka

**Affiliations:** 1 Kabianga University College, P.O. Box 2030-20200, Kericho, Kenya; 2 Department of Medical Physiology, School of Medicine, Moi University, P.O. Box 4606, Eldoret, Kenya; 3 Disease Prevention and Control, Ministry of Public Health, Kenya, P.O. BOX 45335, 00100 (GPO) Nairobi, Kenya; 4 School Natural Resources and Environmental Studies, Karatina University, P.O. Box 1957-10101, Karatina, Kenya; 5 Department of Aquatic Ecology and Ecotoxicology, Institute for Biodiversity and Ecosystem Dynamics, University of Amsterdam, Science Park 904, Amsterdam 1098 XH, The Netherlands; 6 Department of Environmental Biology and Health, School of Environmental Studies, University of Eldoret, P.O. Box 1125, Eldoret, Kenya; 7 Institute of Tropical Medicine and Infectious Diseases (ITROMID), Jomo Kenyatta University of Agriculture and Technology (JKUAT), P.O. Box 62000-00200, Nairobi, Kenya; 8 Tropical Pesticides Research Institute, Division of Livestock and Human Diseases Vector Control, Mosquito Section, Ngaramtoni, Off Nairobi road, P.O. Box 3024, Arusha, Tanzania

**Keywords:** Malaria, Malaria diagnosis, Symptomatic diagnosis, Parasite density, Anaemia, Haemoglobin levels

## Abstract

**Background:**

The commonly accepted gold standard diagnostic method for detecting malaria is a microscopic reading of Giemsa-stained blood films. However, symptomatic diagnosis remains the basis of therapeutic care for the majority of febrile patients in malaria endemic areas. This study aims to compare the discrepancy in malaria and anaemia burdens between symptomatic diagnosed patients with those diagnosed through the laboratory.

**Methods:**

Data were collected from Western Kenya during a follow-up study of 887 children with suspected cases of malaria visiting the health facilities. In the laboratory, blood samples were analysed for malaria parasite and haemoglobin levels. Differences in malaria prevalence between symptomatic diagnosis and laboratory diagnosis were analysed by Chi-square test. Bayesian probabilities were used for the approximation of the malaria and anaemia burdens. Regression analysis was applied to: (1) determine the relationships between haemoglobin levels, and malaria parasite density and (2) relate the prevalence of anaemia and the prevalence of malaria.

**Results:**

The prevalence of malaria and anaemia ranged from 10% to 34%, being highest during the rainy seasons. The predominant malaria parasite was *P. falciparum* (92.3%), which occurred in higher density in children aged 2‒5 years. Fever, high temperature, sweating, shivering, vomiting and severe headache symptoms were associated with malaria during presumptive diagnosis. After conducting laboratory diagnosis, lower malaria prevalence was reported among the presumptively diagnosed patients. Surprisingly, there were no attempts to detect anaemia in the same cohort. There was a significant negative correlation between Hb levels and parasite density. We also found a positive correlation between the prevalence of anaemia and the prevalence of malaria after laboratory diagnosis indicating possible co-occurrence of malaria and anaemia.

**Conclusion:**

Symptomatic diagnosis of malaria overestimates malaria prevalence, but underestimates the anaemia burden in children. Good clinical practice dictates that a laboratory should confirm the presence of parasites for all suspected cases of malaria.

## Background

Despite global efforts to eradicate malaria, the disease burden is increasing worldwide, with almost two million estimated deaths annually [1,2]. The situation is dire in Sub-Saharan Africa (SSA) due to many associated factors, including poverty and ignorance about the disease [3]. The protozoan parasite of the genus *Plasmodium*, which lives in the erythrocytes of the host, is the causative agent of malaria in humans. In many areas of SSA with stable malaria, infestations with *Plasmodium* remain asymptomatic, undetected and untreated [4-6]. Only a small percentage of individuals ever exhibit clinical symptoms. The parasites cause increased haemolysis of the erythrocytes depending on their burden [7,8]. Anaemia occurs due to direct depression of erythropoiesis by malarial infection and actual parasitization of red cells, leading to shortened survival or death of erythrocytes [9]. Therefore, detection of the *Plasmodium* will not only lead to proper treatment of malaria, but also augments the detection of anaemia in the same cohort.

There are now two commonly used methods of malaria diagnosis, especially in SSA: laboratory diagnostic methods and symptomatic diagnosis. In the laboratory diagnostic methods, the blood samples are analysed for *Plasmodium* parasites (normally by trained personnel) using a microscope. It is also possible to identify patients with anaemia based on the haemoglobin counts during laboratory analysis. Electing to use laboratory analysis, however, has its challenges in SSA, since most deaths due to malaria occur at home. Furthermore, functional microscopes are not available in most health facilities frequented by patients [10]. For the symptomatic method of diagnosis, patients are (self) diagnosed based on the symptoms of malaria and given a prescription to control or treat the perceived “malaria” based on symptoms alone. This method is always based on the premise that malaria manifest clear symptoms in the patients. Management of malaria is based on self reported symptoms alone without further laboratory confirmation of the presence of *Plasmodium* parasites [11,12]. The reasons for the symptomatic management of malaria are diverse and range from ease of access to the (sometime unqualified) medical personnel, economic problems, repeat incidences of malaria among patients, high malaria prevalence and failure of the formal health sector [13-16]. Symptomatic diagnosis is less expensive, most commonly used method and is the basis for self treatment. However, the overlapping of malaria symptoms with other tropical diseases impairs its specificity and therefore, encourages the indiscriminate use of anti-malarials for managing febrile conditions in endemic areas. This practice was understandable in the past when inexpensive and well-tolerated anti-malarials were still effective [17,18].

In SSA, there is a limitation of finance for proper health care and low number of qualified medical personnel; there are also fewer hospitals that can adequately cover the population [19]. Surprisingly, hospitals that perform proper laboratory diagnosis are limited to few urban areas. These challenges render laboratory diagnosis for many patients with symptoms of malaria out of reach. While there is a plethora of research on the need for proper malaria diagnosis to control drug resistance and its associated complications, complemented by few research outputs on the role of poor malaria diagnosis, to-date, there has been no study on the magnitude of symptomatic diagnosis of malaria on the epidemiology of malaria and anaemia. The aim of this study was therefore to determine the role of symptomatic diagnosis on the management of malaria and anaemia in malaria endemic population. We hypothesize that patients who undergo symptomatic malaria diagnosis will have lower prevalence of malaria, but with a higher prevalence of anaemia.

## Methods

### Study design and settings

The study participants were obtained from Nandi and Uasin Gishu County, located in the Western part of the Kenya. Nandi County is situated at latitude 34°48’51.70”E- 35°26’04.52” E and longitude 0°06’50.90”S- 0°33’17.78”N. The Uasin Gishu County is located at latitude 35°07’41.63”-35°30’14. 52”E and longitude 0°00’34.73”N- 0°55’39.76”N. The rainfall ranges from 1200 mm to 2000 mm. The area has a moderate malaria prevalence (13-25%) [20]. The economy of the local people is primarily rural subsistence agriculture, including maize, beans, wheat, and cash crop such as tea and pyrethrum. Long rains fall from March through May and short rains from October through November.

This study was conducted between January to December 2010. The epidemiological data on patients were collected from 15 hospitals located in Nandi and Uasin Gishu Counties. They included hospitals in Turbo (n = 1), Kapkangani (n = 1) Kapsabet (2), Ndalat (n = 1), Chepkoilel (n = 1), Kesses (n = 2), Kapseret (n = 2) and Eldoret Town (n = 5). We stationed two research assistants in each of the 15 health facilities to identify the patients attending the facilities for malaria diagnosis and treatment. There were a total of 2022 children initially enrolled in the study based the number of visits to the hospitals. However, we managed to successfully follow 887 who had sought treatment after symptomatic diagnosis during the one year period of the current study. Following the slide examination by the project technician, confirmed malaria cases were referred for treatment with malaria drugs provided by the research team.

### Symptomatic diagnosis of malaria

Each person selected for the current study population was clinically examined by two project physicians. The entry into the study population was made on the basis of observed elevated temperature (>37.6°C). Other symptoms that are associated with malaria that were considered were: fever, profuse sweating, shivers, vomiting, severe headache, dehydration, nausea, diarrhoea, convulsions, jaundice, myalgia, backache, and joint pains. A clinical history of symptoms was taken, including length of illness. The duration, combination, and sequence of symptoms were noted. Other personal data were collected from the respondents using structured questionnaires.

### Laboratory methods

All the laboratory-based analyses of the study participants were conducted Moi Teaching and Referral Hospital (Accreditation number NCST/NBC/AC/0612). Microscopic analysis was done to identify the types of malaria parasite and their respective densities. For malaria parasites analyses; blood samples were collected by the standard finger-prick method and thick and thin smears prepared on labelled slides. The smears were allowed to air dry, then fixed in methanol and stained in 4% Giemsa for 30 minutes. The stained smears were afterwards examined using the magnification of × 1,000 (oil immersion) to identify and count the parasite species. For quality control, 10% of the negative samples and 20% of the positive samples were re-examined by at least two microscopist during the study. Results showing large discrepancies (larger than 5% confidence interval) were referred to the third expert for analysis. Parasite density was scored against 300 leukocytes in positive slides. Parasite densities were converted to a number of parasites per microliter of blood, assuming a leukocyte count of 8,000 cells/μL [21].

The WHO definition of anaemia [22] was used during the study, hereby defined as Hb < 11 g/dL, and categorized as mild (Hb 8.0‒10.9 g/dL), moderate anaemia (Hb 5.1‒7.9 g/dL) and severe anaemia (< 5 g/dL). The haemoglobin level of study subjects was measured to the nearest 0.1 g/dL using Hemocue (HemoCue® HB 301 System, Ängelholm, Sweden SE-262 23).

### Statistical analysis

Data was entered and validated using Epi-Info™ version 6.04d software [23]. The data collected were analysed using SPSS (version 17.0, SPSS Inc. Chicago, IL, USA). Parasite density was presented as mean ± SEM. Age specific differences in parasite densities were analysed using One-Way ANOVA. Differences in malaria prevalence of patients based on symptomatic and laboratory diagnosis of the same cohort were analysed using the Chi-square (*χ*^2^). Bayesian probabilities were used to estimate the malaria and anaemia burden in the presumptively and laboratory diagnosed malaria cases. The relationships between: haemoglobin levels and malaria parasite density as well as between the prevalence of anaemia and prevalence of laboratory diagnosed malaria in children were assessed by regression analysis. For all statistical tests, *p* < 0.05 was considered significant.

### Ethical review and informed consent

The study protocol was reviewed and approved by the institutional review boards of the Kenya Medical Research Institute (Nairobi, Kenya) and the Moi University Ethical Review Committee (Reference: IREC/2010/18; FAN: IREC 000517). All survey participants provided written informed consent. For children, the parents provided written informed consent.

## Results

Of 2022 children initially enrolled; 887 finished the full follow-up and were included in the follow up survey. Pertinent characteristics of the study subjects who completed the follow up study are presented in Table 1. In our study, children < 6 months of age were lower in proportion in our samples because their parents refused to divulge some information about them due to the cultural norms in some of the homesteads, therefore some of the data were not used in the current study as they were suspected to be inaccurate. Episodes of previous malaria cases also manifested from the responses from the parents of study subjects.

**Table 1:** Characteristics of the study subjects (n = 887)

**Characteristics**		**n (%)**
Age (Months)	< 6	34 (3.8)
	6 – 12	223 (25.1)
	12.1 - 24	202 (22.8)
	24.1 - 60	178 (20.1)
	60.1 - 144	108 (12.2)
	> 144	142 (16.0)
Gender	Male	451 (50.8)
	Female	436 (49.2)
Sleep under bed nets	Yes	581 (65.5)
	No	224 (25.3)
	Not sure	82 (9.2)
Number of malaria treatments	None	44 (5.0)
	Once	189 (21.3)
	Occasionally	456 (51.4)
	Frequently	198 (22.3)

**Figure 1:**
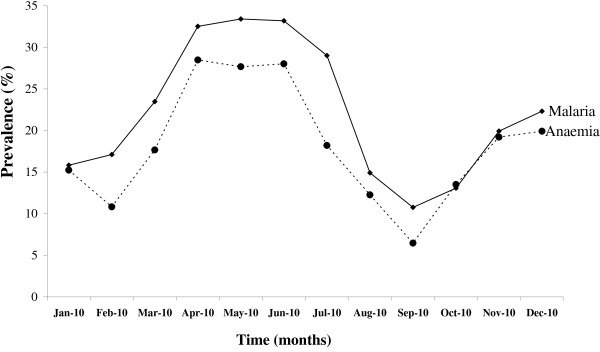
Prevalence of malaria and anaemia during the year 2010.

**Figure 2:**
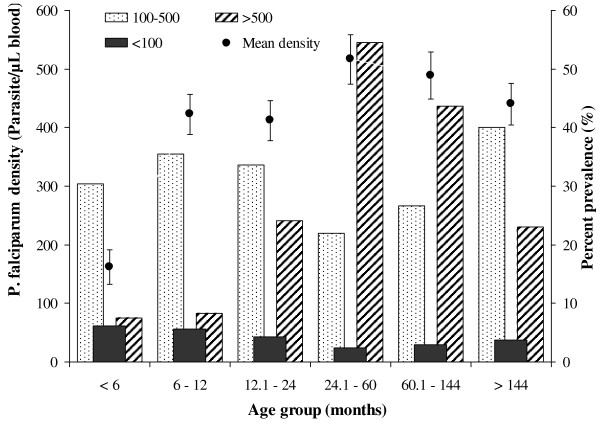
**Age specific mean density of *Plasmodium falciparum *** **and prevalence of different parasite densities in the study respondents.**

We conducted a study of the prevalence of malaria and anaemia in our initial sample of 2022 in the study area in the year 2010 (Figure 1). The prevalence of malaria ranged from 10% to 34%, the peak incidences being reported during heavy rainfall seasons between April to June. The prevalence of malaria was high among the respondents aged 2‒5 years. There was a significant positive correlation between prevalence of anaemia and the prevalence of malaria among the study subjects (R^2^ = 0.7821, *p* = 0.0001). The predominant species of infection were *P. falciparum* (92.3%), 4.6% of all infections were *P. malariae*, 3.1% were mixed infections (*P. falciparum* and *P. malariae*). Age specific mean density of *P. falciparum* and prevalence of different parasite densities among study respondents is presented in Figure 2. There were significant age specific differences in the density of *P. falciparum* among study respondents (*p* = 0.0008). The highest mean density of *P. falciparum* occurred in children aged 2‒5 years (516.4 parasite/μL blood) followed by those aged 5‒12 years (489.2 parasites/μL blood) while those aged < 6 months had the least parasite density in blood smears (162 parasites/μL blood). In terms of *P. falciparum* densities, the study area had discernable age-specific variations in malaria density (*p* = 0.0004) (Figure 2). Malaria parasite density > 500 parasites/μL blood increased in patients aged < 6 months (7%) to 5 years (50%) and thereafter declined. Nevertheless, subjects with parasite density < 100 parasites/μL blood in the population was low (rarely exceeding 5%), with no discernable trends among the respondents.

Overall, 99% (n = 887) of the study subjects reported severe headache, 855 individuals (97.5%) reported high temperature, 820 (92.4%) had fever, 819 (92.3%) had sweated, 818 (92.2%) shivered while 798 (90%) reported vomiting as signs of malaria (Table 2). Other symptoms such as dehydration, nausea, diarrhoea, and joint pains remotely suggested the presence of malaria and/or other ailments, which required over the counter drugs to control without necessarily conducting medical tests on the exact nature of the ailment present. Presence of anaemia was reported by only those patients who had prior knowledge of the disease, which were confirmed from their previous visits to the medical centres in the area. There was remarkably little age-specific variation in these frequencies except for children < 6 months with high fever (98.4%) and temperature (98.3%).

**Table 2:** Commonly reported symptomatic features of malaria during presumptive diagnosis

**Symptomatic feature**	**Frequency (n = 887)**	**Percent**
Fever	820	92.4
High temperature	855	97.5
Sweating	819	92.3
Shivering	818	92.2
Vomiting	798	90.0
Severe headache	878	99.0
Some dehydration	357	40.2
Severe dehydration	201	22.7
Nausea	426	48.0
Diarrhoea	389	43.9
Convulsions	249	28.0
Jaundice	199	22.4
Myalgia	175	19.7
Backache	175	19.7
Joint pains	141	15.9
Anaemia	25	2.8

Up to 60.4% of the study population did not have anaemia. However, 29.8% of the sample had mild anaemia, 7.0% moderate anaemia, while 2.8% of the sample reported severe anaemia. During our follow up study of the study population who apparently had malaria after symptomatic malaria diagnosis, between 31.2-41.0% were confirmed positive for malaria through microscopic analysis, and 20.7-35.3% were found to be anaemic (Table 3). For all the age group above 12 months, all cases of symptomatic diagnosis, the prevalence of malaria through laboratory confirmation ranged between 34.9% to 41%, albeit with a higher prevalence of anaemia than malaria from the same symptomatic diagnosed children (*p* = 0.0002).

**Table 3:** Differences in the prevalence of malaria in patients based on presumptive and laboratory diagnosis in the same cohort

**Age (Months)**	**The prevalence of malaria based on presumptive diagnosis**	**Prevalence of malaria after laboratory diagnosis**	**P-value**	**Prevalence of anaemia after laboratory diagnosis**
< 6 (n = 34)	70.6	31.2	< 0.001	19.6
6 – 12 (n = 223)	47.1	34.8	< 0.001	20.7
12.1 – 24 (n = 202)	73.8	35.4	< 0.001	35.3
24.1 - 60 (n = 178)	66.3	41.0	< 0.001	30.3
60.1 – 144 (n = 108)	72.2	38.8	< 0.001	34.7
> 144 (n = 142)	63.4	34.9	< 0.001	30.7

Reported also are prevalence of anaemia in the same age group after laboratory analysis. No diagnosis of anaemia was done during presumptive diagnosis.

**Figure 3:**
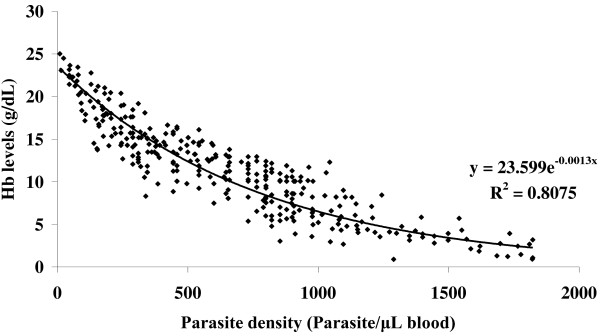
Regression of haemoglobin levels (g/dL) against malaria parasite density (parasite/μL blood) in the respondents in the study area.

**Figure 4:**
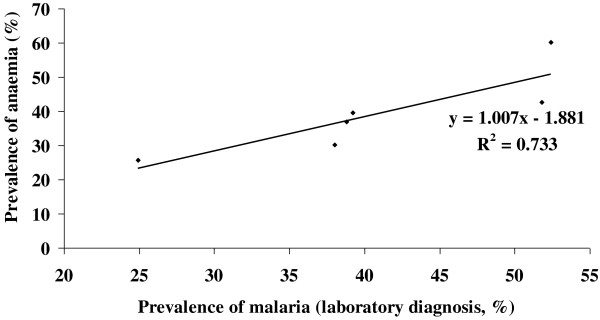
Linear regression between prevalence of anaemia and the prevalence of malaria in laboratory diagnosed patients seeking treatments for malaria.

Overall regression of haemoglobin levels against malaria parasite density among the respondents in the study area is depicted in Figure 3. There was a significant (*p* = 0.0019) negative correlation between Hb levels and parasite density among the study subjects, which exhibited an exponential decrease in Hb levels with increasing malaria parasite density. Additionally, there was a significant (*p* = 0.0021) positive linear association between prevalence of anaemia and prevalence malaria in the laboratory diagnosed children based on the age groups of the study subjects (Figure 4).

The Bayesian probabilities showing the relationships between the probability of malaria burden through presumptive diagnosis and the probability of malaria and anaemia after confirmatory laboratory test revealed several information (Table 4). First, among the patients presumptively diagnosed and presumed to have malaria (64.7%), lower percentage of these individuals were confirmed to have malaria after laboratory diagnostic test (31.3%) with an odds ratio for correct prediction of malaria after presumptive diagnosis being 57.1% while the odds ratio of correctly predicting anaemia in the same cohort being only 3.4%. As for laboratory analysis, 64.7%, of the patients who were presumptively diagnosed and presumed to have malaria, 31.3% were actually confirmed to have the disease and 19.7% were found to have anaemia. The odds ratio of correct prediction of malaria and anaemia burdens following laboratory tests was 99.4% and 93.2% respectively.

**Table 4:** Bayesian probability of correct estimation of malaria and anaemia prevalence during presumptive and laboratory diagnosis

	**Disease prevalence in %**		**Probability of correct estimation of the disease burden**	
Type of malaria diagnostic	Malaria	Anaemia	Malaria	Anaemia
Presumptive diagnosis	64.7	Not tested	0.571	0.034
Laboratory test	31.3	19.7	0.994	0.932

## Discussion

Prior to this study, we found out that out of 25,530 patients who had visited the health centres for malaria treatments in the study area between the years 2001 and 2010, 58.3% who sought treatment through symptomatic diagnosis, later sought laboratory diagnosis and subsequent treatment for malaria after the increased severity of the disease (unpublished medical records from 15 health facilities). The remaining 41.7% were symptomatically diagnosed and treated for malaria without seeking any intervention from the hospital. Few and mostly inconsistent records are available for laboratory confirmation of the malaria prevalence among the presumptively diagnosed patients without any evidence that diagnoses of anaemia among the patients were ever conducted in patients seeking malaria treatments. Therefore, the aim of this study was to determine the variance between the prevalence of malaria and anaemia in patients following presumptive diagnosis and treatment compared to those seeking laboratory diagnosis and detection of the *Plasmodium* before treatment from a follow-up of the same patients. We hypothesized that the patients who undergo symptomatic malaria diagnosis will have lower prevalence of actual malaria cases with higher prevalence of anaemia. During the study, we ascertained that the sensitivity and specificity of our microscopist were above 95% through frequent training and consultation. Strict adherence to procedures for slide preparation and staining [24] ensured the production of clear, well stained slides, thereby reducing errors due to artefacts.

In Kenya, malaria transmission patterns are classified based on transmission intensity as endemic, and unstable endemic zones and epidemic zones [25,26]. From our survey of the households in the year 2010, the prevalence of malaria ranged from 10 to 34%, which peaked during heavy rainfall seasons, suggesting that the area is clearly malaria mesoendemic area in which the prevalence rate of malaria is 11–50% during most of the year and experience irregular rapid increases in malaria incidences usually related to seasons and population movements. Most populations in the epidemic zones are not immune and therefore, all age-groups are at risk of severe malaria infection [27].

*Plasmodium* causes severe complications as cerebral malaria, severe anaemia, acute renal failure, hypoglycaemia and pulmonary infection. In the current study, microscopic examination of blood smears was used to confirm *P. falciparum* as the most prevalent (92.3%) while *P. malariae* was 3.1%, similar to previous findings [28-30]. The two characteristic features that actually separate *P. falciparium* from the other human malaria is the ability to attack erythrocytes of all ages, causing high parasitaemia and enhanced growth and the capability to adhere to vascular endothelium through sequestration [31]. The highest mean density of *P. falciparum* among children aged 2‒5 years, perhaps explain why this age bracket is highly affected by the occurrence of malaria in many countries of SSA. The lowest mean parasite density among children aged < 6 months may be due to higher frequency of insecticide treated mosquito bed nets usage usually provided for the mothers immediately after birth [28] or may be related to the passive immunity acquired via the maternal antibodies [32].

During the study, most of the study subjects reported presence of fever, high temperature, sweating, shivering, vomiting and severe headache as a precursor of malaria, despite the obvious sign that these symptoms may also indicate the presence of other diseases. This method is likely to underestimate the occurrence of other diseases in the human populations. In the present study, patients who suspected they were suffering from malaria did not seek medical laboratory confirmation for malaria parasites, and neither did a test for the presence of anaemia. However, overall mild anaemia had been about 30%, moderate anaemia was 7.0%, while severe anaemia was 2.8% indicating that 40% of the patients had anaemia, which would remain undetected through symptomatic diagnosis. We also established that children who were symptomatically diagnosed, higher percentage had anaemia, with the highest differences being observed among children aged between 2‒5 years due to the high density of *P. falciparum* parasites. These observations may suggest that the *P. falciparum* parasites are undetected during symptomatic diagnosis and are causing increased haemolysis of parasitized erythrocytes leading to a higher prevalence of anaemia [4,7].

We confirmed that indeed there was a negative correlation between Hb levels and parasite density among the study subjects, suggesting direct depression or haemolysis of the erythrocytes by *Plasmodium* infection [9]. Again, the association between the prevalence of anaemia and prevalence of laboratory diagnosed malaria in the study subjects was found to be significant. These outcomes are important in several ways. First, suspicion of malaria is always based on symptoms of the disease and therefore is the sole basis for seeking treatment in countries where malaria is endemic. This ordinarily results in all patients with fever and no apparent causes of disease being treated for malaria. Although this approach can immediately identify most patients who need malaria treatment, it also is likely to misclassify many who do not, leading to those with other diseases receiving malaria treatment. While this might have been accepted in the past when malaria was treatable with affordable and relatively safe drugs, it is not acceptable today. A diagnosis based on symptoms alone has very low specificity. As a result, malaria can be over-diagnosed considerably, while other diseases are overlooked and not treated in a timely manner. Given the role of malaria parasites in haemolysis of the erythrocytes, this may contribute to problems of increased anaemia in the population. Indeed, based on the Bayesian probabilities, we established that during presumptive diagnosis of malaria, the probability of a correct diagnosis and confirmation of malaria as compared to the laboratory test was generally low. Furthermore anaemia was undetected in the malaria patients seeking presumptive diagnosis without laboratory confirmation, which ultimately increase anaemia burden among the patients.

Symptomatic diagnosis is imprecise, but remains the basis of therapeutic care for most of the febrile patients in malaria endemic areas, where laboratory support is often out of reach [11,18]. However, malaria rapid diagnostic tests (RDTs) are an alternative diagnostic method for endemic regions, where microscopy has not been implemented, as well as for non-endemic countries, where they are able to complement microscopy in screening febrile patients [33-35]. Thus, RDTs are seen as a way of extending parasitological diagnosis of malaria to peripheral health facilities without microscopy. The RDTs detect parasite antigens from a peripheral blood sample with reasonable sensitivity and specificity and can be used at peripheral health facilities with minimal training [36]. Several of these RDTs showed high efficiency for the diagnosis of malaria in different conditions [36-40]. One of the drawbacks of these devices is the quality of tests compared to microscopy and their practicability for local staff in remote areas [41]. Moreover, the current market price of an RDT in developing countries is about U.S.$0.55–U.S.$1.50 (depending on the number of targeted species and the order quantity), compared with microscopy at U.S.$0.12–$0.40 per malaria smear [33]. In malaria endemic countries, RDTs may be relied upon to contribute to rationalization of treatment of febrile illness in areas where microscopy diagnosis is not available, not reliable or not performed immediately.

## Conclusion

This study provides clinical evidence that symptomatic diagnosis of malaria overestimates prevalence of malaria, but underestimates the prevalence of anaemia in children. Despite primary health care strategies for the reduction of mortality and morbidity in children resulting from malaria, malaria induced anaemia still occur frequently. This perhaps suggests that additional and urgent measures are needed to in combating malaria and its complications of which anaemia forms a major part. Although symptomatic diagnosis and treatments of malaria have been considered reasonable in resource-poor settings with high malaria transmission where laboratory infrastructure is inadequate, the contemporary level of misdiagnosis is unsustainable particularly resulting in overestimation of malaria burden while increasing the incidences of anaemia among the patients. So, sound clinical practice dictates that a laboratory should confirm the presence of parasites for all suspected cases of malaria, which can be achieved through RDTs and/or microscopic analysis.

## Competing interests

All authors declare that they have no competing interests.

## Authors’ contributions

JKC conceived and designed the study; MAGN, JKC and WA collected data from the field. MMN, FE provided logistical support and vital equipment during research design and data collection. JKC, EO-O, FA, WA, JK, CK-W, EJK and OO participated in drafting and revising the manuscript. WA also provided most of the research materials. EO-O analysed the data. All authors have read and approved the final manuscript.

## Pre-publication history

The pre-publication history for this paper can be accessed here:

http://www.biomedcentral.com/1471-2458/14/332/prepub
